# Acute Kidney Injury and Electrolyte Imbalances Caused by Dapagliflozin Short-Term Use

**DOI:** 10.3390/ph17040420

**Published:** 2024-03-26

**Authors:** António Cabral Lopes, Olga Lourenço, Sandra Morgado, Andreia Gaspar, Idalina Freire, Inês Eusébio, João Ribeiro, Mafalda Silva, Marta Mendes, Olímpia Fonseca, Rute Duarte, Manuel Morgado

**Affiliations:** 1 Pharmaceutical Services of Local Health Unit of Guarda (ULS da Guarda), 6300-035 Guarda, Portugal; 2 FCS-UBI, Faculty of Health Sciences, University of Beira Interior, 6200-506 Covilhã, Portugal; olga@fcsaude.ubi.pt (O.L.); srolo@chcbeira.min-saude.pt (S.M.); ifreire@fcsaude.ubi.pt (I.F.); jcribeiro@chcbeira.min-saude.pt (J.R.); olimpia.fonseca@chcbeira.min-saude.pt (O.F.); mmorgado@fcsaude.ubi.pt (M.M.); 3 CICS-UBI, Health Sciences Research Centre, University of Beira Interior, 6200-506 Covilhã, Portugal; 4 Pharmaceutical Services of Local Health Unit of Cova da Beira (ULS Cova da Beira), 6200-251 Covilhã, Portugal; andreia.gaspar@chcbeira.min-saude.pt (A.G.); ines.eusebio@chcbeira.min-saude.pt (I.E.); mmaricoto@chcbeira.min-saude.pt (M.S.); mdmendes@chcbeira.min-saude.pt (M.M.); rute.duarte@chcbeira.min-saude.pt (R.D.)

**Keywords:** dapagliflozin, electrolyte balance, renal function, T2DM

## Abstract

Dapagliflozin, a sodium–glucose cotransporter 2 inhibitor (SGLT2i), has shown demonstrated benefits for renal and cardiovascular outcomes in large clinical trials. However, short-term concerns regarding its impact on renal function and electrolyte balance exist. This study aimed to evaluate the short-term effects of dapagliflozin on renal function and electrolyte balance in patients newly prescribed the medication. A retrospective analysis of 246 patients who initiated dapagliflozin therapy was conducted. Serum creatinine, sodium, and potassium levels were measured at baseline (before dapagliflozin) and 5–8 days after initiation (endpoint). A Wilcoxon signed-rank test, Pearson’s chi-square test, and Fischer’s exact test were used for the data analysis. Glycemia and sodium levels were significantly higher at the baseline compared to the endpoint (*p* < 0.001). Conversely, creatinine and potassium levels were significantly higher at the endpoint than at the baseline (*p* < 0.001). The prevalence of hyponatremia and hyperkalemia were increased at the endpoint (17.5% vs. 10.2% and 16.7% vs. 8.9%, respectively). Although not statistically significant, a trend towards increased hyponatremia with the co-administration of furosemide was observed (*p* = 0.089). No significant association was found between potassium-sparing medications (*p* > 0.05) and hyperkalemia, except for angiotensin receptor blockers (*p* = 0.017). The combination of dapagliflozin and furosemide significantly increased the risk of acute kidney injury (AKI) at the endpoint (*p* = 0.006). Age, gender, and chronic kidney disease status did not significantly influence the occurrence of AKI, hyponatremia, or hyperkalemia (*p* > 0.05). These findings emphasize the importance of the close monitoring of renal function and electrolyte balance, particularly in the early stages of dapagliflozin therapy, especially in patients receiving diuretics or renin–angiotensin–aldosterone system inhibitors.

## 1. Introduction

Sodium–glucose cotransporter-2 inhibitors (SGLT2is) are a class of antidiabetic drugs (ADs) used for the treatment of type 2 diabetes mellitus (T2DM). They act by impeding renal glucose reabsorption and promoting urinary glucose excretion, through the inhibition of the glucose high-capacity transporter SGLT2 located in the proximal convoluted tubule. This distinctive mechanism of action operates independently of insulin and is contingent upon blood glucose levels, concurrently enhancing sodium elimination [1,2,3,4]. This unique mechanism of action complements that of other classes of ADs, allowing for its use in combination therapy, including with insulin [5]. SGLT2is have a modest natriuretic effect that may lower blood pressure; however, due to other compensatory mechanisms, this effect is transient. The blood pressure-lowering effect of SGLT2is is likely influenced by other factors, including weight loss and diuresis [4,6].

Dapagliflozin has emerged as one of the most widely prescribed SGLT2is globally, with its pharmacological attributes and clinical applications having undergone extensive scrutiny. Nonetheless, the research examining its short-term impact on renal function and electrolyte balance in real-world settings remains limited [7,8,9,10,11,12]. Recent studies, including those involving dapagliflozin, have showcased significant reductions in hospitalizations due to heart failure (HF), cardiovascular (CV) events, and mortality [13,14]. In response, the American College of Cardiology (ACC) issued the “2020 Expert Consensus Decision Pathway on Novel Therapies for CV Risk Reduction in Patients with T2DM”, specifically advocating for the use of SGLT2is in patients with or without T2DM, particularly those with HF and reduced ejection fraction (HFrEF) [15]. The vasodilatory effects of SGLT2is, attributable to afferent arteriolar vasoconstriction mediated by adenosine-induced myogenic activation, contribute to reduced intraglomerular pressure and glomerular filtration rate, thereby fostering renal and cardiovascular protective effects. These mechanisms include natriuresis, blood pressure reduction, and the attenuation of oxidative stress and fibrosis [16,17]. The DAPA-CKD trial showed that among patients with chronic kidney disease (CKD), regardless of the presence or absence of T2DM, the risk of a combination of sustained decline in the estimated GFR of at least 50%, end-stage kidney disease, or death from renal or cardiovascular causes, was significantly lower with dapagliflozin than with a placebo [18,19]. Despite their documented benefits, discrepancies persist regarding the influence of SGLT2is on urinary sodium concentration and output [20,21,22,23,24]. Additionally, heightened susceptibility to electrolyte imbalances, notably hyponatremia and hyperkalemia, have been noted in patients with T2DM, particularly those with concomitant CKD, necessitating vigilant monitoring during treatment with drugs such as angiotensin-converting enzyme inhibitors (ACEis) and angiotensin receptor blockers (ARBs) [25,26,27,28]. The concomitant use of diuretics with other medications presents a potential risk of acute kidney injury (AKI), primarily characterized by tubular epithelial degeneration [29]. While certain studies have suggested that furosemide may exacerbate structural and functional kidney damage in cases of prior renal failure, its precise impact remains unclear [30,31].

Managing patients with CKD poses significant challenges, particularly concerning the risk of hydroelectrolytic disorders. As renal function declines, the ability to maintain water homeostasis diminishes, rendering CKD patients more susceptible to significant alterations in natremia and hyperkalemia [32,33,34,35]. Patients with CKD face an increased risk of hyponatremia due to impaired urine concentration ability, a common electrolyte disorder exacerbated by aging. Age is a strong independent risk factor for the occurrence of hyponatremia, which is associated with greater susceptibility to it and increased morbidity, underscoring the importance of prevention and management, especially in geriatric populations. Similarly, hyperkalemia can be potentially fatal in patients with CKD. While reduced renal function is the primary cause, other factors, such as insulin deficiency, excessive potassium supplementation, and renal potassium excretion decreases from medications like ACEis, ARBs, or potassium-sparing diuretics, can exacerbate or cause hyperkalemia [36,37,38,39,40].

This study aimed to assess the short-term effects of dapagliflozin on renal function and electrolyte balance in hospitalized patients receiving the drug for the first time. We compared our findings with adverse drug reaction (ADR) profiles reported in the Eudravigilance (EV) database for a similar level of care. Through a comprehensive assessment and comparison with real-world data, we aimed to provide valuable insights that could guide patient management strategies and optimize treatment outcomes.

## 2. Results

### 2.1. Sample Characterization

**Figure 1:**
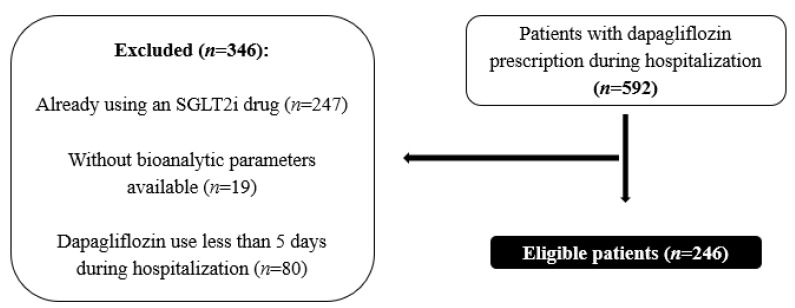
Sample selection.

Initially, a total of 592 patients with a prescription for dapagliflozin were identified. Subsequently, 346 patients were excluded from this study for various reasons: existing use of an SGLT2i (*n* = 247), unavailability of bioanalytic parameters necessary for the intended analysis (*n* = 19), or dapagliflozin use of less than 5 days during hospitalization (*n* = 80) (Figure 1).

Ultimately, 246 patients met the inclusion criteria, of whom 123 were male (50.0%). The comorbidities included a previous diagnosis of T2DM in 149 patients (60.6%), HBP in 185 (75.2%), HF in 114 (46.3%), and CKD in 64 (26.0%) (Table 1). Notably, 174 patients (70.7%) were concomitantly using furosemide, 94 (39.2%) spironolactone, 88 (35.8%) an ACEi, 86 (35.0%) an ARB, and 77 (31.7%) received potassium supplementation (Table 1).

### 2.2. Effects of Dapagliflozin on Renal Function

According to the KDIGO Guidelines, AKI is defined as when blood creatinine increases by 0.3 mg/dL (26.5 μmol/L) or more in 48 h, or rises at least 1.5-fold from the baseline within 7 days [41].

Blood creatinine levels before dapagliflozin administration were significantly lower at the baseline (mean rank = 112.01) than at the endpoint (mean rank = 130.08) (*p* = 0.0001, Wilcoxon signed-rank test) (Table 2). 

There was not a statistically significant association between gender and AKI at the endpoint (*p* = 0.878, Pearson’s chi-square test) or between age and AKI (*p* = 0.232, Pearson’s chi-square test) (Table 2).

Furthermore, a logistic regression model (Forward LR method) with the independent variables being age, disease, and the drug used, revealed that only furosemide significantly contributed to an increase in the incidence of AKI (*p* = 0.008), consistent with the findings presented in Table A1.

### 2.3. Effects of Dapagliflozin on Electrolyte Balance

Blood sodium levels were significantly higher before dapagliflozin administration (mean rank = 117.36) than at the endpoint (mean rank = 107.55) (*p* = 0.0009, Wilcoxon signed-rank test), as was the percentage of patients with hyponatremia (*p* = 0.0146, Pearson’s chi-square test) (Table 3). Conversely, blood potassium levels were significantly lower before dapagliflozin administration (mean rank = 98.64) than at the endpoint (mean rank = 127.82) (*p* < 0.0001, Wilcoxon signed-rank test), as was the percentage of patients with hyperkalemia (*p* = 0.0002, Pearson’s chi-square test) (Table 4).

The use of furosemide did not show a statistically significant association with the occurrence of hyponatremia at the endpoint (*p* = 0.089, Pearson’s chi-square test). Regarding hyperkalemia occurrence at the endpoint, there was no statistically significant association found with potassium supplementation (*p* = 0.243), ACEis (*p* = 0.096), or spironolactone administration (*p* = 0.149). However, a statistically significant association was observed with the administration of ARBs and dapagliflozin (*p* = 0.017) (Table 4).

### 2.4. Comparisons with Adverse Drug Reaction Profiles Reported in the Eudravigilance Database

From 1 January 2023 to 31 December 2023, a total of 2666 Individual Cases Safety Reports (ICSRs) were reported based on a suspicion of dapagliflozin use. Of those, 1517 (56.9%) were reported for male patients, 1033 (38.7%) for female patients, and 116 (4.4%) did not specify the sex. The age distribution indicated that 1017 cases (38.1%) were in the age group 65–85 years, 612 (23.0%) were in the age group 18–64 years, 199 (6.5%) in the age group > 85 years, and 833 (31.2%) had no specified age group. Among the reported cases, 1425 (53.5%) were for the treatment of diabetes mellitus, 237 (8.9%) for CKD, and 502 (18.9%) for HF (Table 5). 

Regarding the most reported cases by system organ classes, 394 cases (14.8%) were related to renal and urinary disorders, 126 (4.7%) to cardiac disorders, 314 (11.8%) to gastrointestinal disorders, and 595 (22.3%) to infections (Table 5).

A statistical analysis revealed no statistically significant difference between the concomitant use of furosemide and AKI (*p* = 0.770, Pearson’s chi-square test) or hyponatremia (*p* = 0.066, Fisher exact test), nor between the concomitant use of an ACEi (*p* = 0.335, Fisher exact test) or ARA (*p* = 1.000, Fisher exact test) and hyperkaliemia. However, there was a statistically significant difference between the concomitant use of spironolactone and hyperkaliemia (*p* = 0.002, Fisher exact test) (Table 5). 

## 3. Discussion

Studies have consistently demonstrated the efficacy of dapagliflozin for improving glycemic control in patients with T2DM. Our findings align with these previous reports, showing a significant reduction in blood glucose levels within the first 5 to 8 days of dapagliflozin administration. While the glucose-lowering effect of dapagliflozin can begin after just a single dose, clinical significance may take up to a week to manifest [42]. The observed reduction in glycemia within the first week of dapagliflozin initiation underscores its rapid onset of action and highlights its potential as an early treatment option for patients with T2DM.

Observational studies have corroborated the results reported by placebo-controlled clinical trials, showing a reduction in AKI episodes in T2DM patients treated with SGLT2is compared to alternative glucose-lowering therapies. SGLT2is are increasingly prescribed to non-diabetic patients with CKD, as these agents prevent a decline in renal function by reducing glomerular hypertension, regardless of their effect on glycemic control [43,44]. Our study observed a notable increase in blood creatinine levels post-dapagliflozin administration, suggesting potential renal impairment in a subset of patients. The initiation of an SGLT2i often leads to an initial decline in the estimated glomerular filtration rate (eGFR), followed by a partial recovery over time [45,46]. These initial declines, or dips, were typically seen 2–4 weeks after starting an SGLT2i, with a subsequent partial recovery of the eGFR curve at week 12 [13,18,47]. Some authors have suggested that modest decreases in the eGFR should not encourage clinicians to discontinue treatment with SGLT2is. However, in our study, a significant percentage of patients showed AKI or a worsening of pre-existing CKD [48]. Our findings raise concerns regarding renal function, emphasizing the need for the close monitoring of renal parameters in clinical practice, particularly in the first few weeks after starting dapagliflozin. The transient nature of these declines and their subsequent recovery over time have been documented in major outcome studies of SGLT2is, suggesting a complex interplay between drug effects and renal physiology. Furthermore, the combination of SGLT2is and loop diuretics like furosemide may increase the risk of AKI, as observed in our study.

SGLT2is exert significant effects on sodium and glucose reabsorption in the proximal tubules, leading to increased renal glucose and sodium excretion [49]. Our findings reveal a decrease in blood sodium levels following dapagliflozin administration, potentially related to enhanced renal sodium excretion. While previous studies have suggested transient reductions in extracellular fluid volume with SGLT2i use [50,51], our results underscore the importance of monitoring natremia to prevent neurological complications [52,53]. Interestingly, concomitant furosemide use did not appear to exacerbate hyponatremia in our study cohort. Although corrected sodium levels for hyperglycemia are a better predictor of clinical outcomes than measured sodium levels among patients with extreme hyperglycemia (≥500 mg/dL) [54,55], in our study only two patients had blood glucose levels higher than 500 mg/dL: one patient at the baseline (522 mg/dL) and another at the endpoint (908 mg/dL). Therefore, in this study, we considered measured serum sodium levels without any correction relative to blood glucose values.

Similarly, SGLT2is have been associated with a reduced risk of severe hyperkalemia in high-risk T2DM patients or those with CKD, without increasing the risk of hypokalemia [56,57,58,59]. However, our findings indicate a higher incidence of hyperkalemia at the endpoint, emphasizing the need for careful potassium monitoring, especially in patients receiving concurrent ARBs. 

An analysis comparing the data from the ICSRs to the findings from our hospitalized patient cohort offers valuable insights into the real-world safety profile of dapagliflozin. While our study focused on the short-term outcomes of hospitalized patients, the ICSR data encompassed a broader spectrum of patient experiences over a longer timeframe. Interestingly, the incidence of adverse events reported in the ICSRs, such as renal and urinary disorders, cardiac disorders, gastrointestinal disorders, and infections, underscores the multifaceted nature of dapagliflozin’s safety profile. Although direct comparisons between the two datasets must be interpreted cautiously due to the inherent differences in methodology and patient populations, the alignment of key findings, such as electrolyte disturbances and acute kidney injury, lends support to our observations. 

Patients with CKD are particularly susceptible to electrolyte disturbances, including hyponatremia and hyperkalemia [45,60,61]. While our study did not find a significant association between dapagliflozin use and electrolyte imbalances in CKD patients, the management of these comorbidities remains a clinical challenge. Continuous surveillance of renal function and electrolyte levels is essential in this patient population to mitigate the risk of adverse events and optimize treatment outcomes.

While this retrospective study offers valuable insights, it is important to acknowledge its limitations. The data analysis was not originally intended for research purposes, potentially leading to the omission of certain variables and the presence of selection biases. The limited sample size, coupled with its homogeneity, as the patients were exclusively drawn from two hospitals in the interior region of Portugal with an older population compared to the rest of the country, restricts the generalizability of our findings. Additionally, the lack of daily analytical data for the patients limited our ability to establish a cause–effect relationship with precision. Furthermore, the data obtained from the ICSRs did not always contain detailed information, such as the concomitant medications or age group, limiting the depth of our conclusions. However, despite these limitations, our study provides real-world insights into the clinical implications of dapagliflozin therapy. By capturing data from routine clinical practice, we aimed to bridge the gap between controlled clinical trials and everyday patient care. This approach could be used to inform evidence-based decision making and contribute to an enhancement of the quality of diabetes management in healthcare settings.

## 4. Materials and Methods

### 4.1. Study Design and Sampling

We conducted a retrospective study involving adult patients, both with or without T2DM, who received their first prescription for dapagliflozin during hospitalization. Patients who had not previously taken any SGLT2i, who had taken dapagliflozin for a minimum of 5 consecutive days, and for whom the bioanalytical parameters under study were available were included. Therefore, the inclusion criteria were as follows: (1) age ≥ 18 years; (2) patients (with T2DM or not) who had never taken an SGLT2i; (3) patients who took dapagliflozin for the first time for at least 5 consecutive days during the period of hospitalization; and (4) patients for whom the bioanalytical parameters under study (blood glucose, creatinine, sodium, and potassium levels) were available both before dapagliflozin prescription (baseline) and 5 to 8 days after prescription (endpoint). The exclusion criteria were as follows: (1) age < 18 years; (2) patients who were already taking an SGLT2i prior to hospitalization; (3) patients who took dapagliflozin during hospitalization for less than 5 days; and (4) patients who started taking dapagliflozin during hospitalization but for whom the bioanalytical values under study were not available. The study period encompassed 23 months, from 1 September 2021 to 31 July 2023, at the Local Health Unit of Guarda and 30 months, from 1 January 2021 to 30 June 2023, at the Local Health Unit of Cova da Beira. Ethics approval was obtained from the Ethics Committee of the Local Health Unit of Guarda (SFTSS-REQ-22021; approval date: 3 April 2023) and the Local Unit Health of Cova da Beira (35/2023; approval date: 29 May 2023).

To estimate the prevalence of short-term renal dysfunction and electrolyte imbalance among the patients receiving dapagliflozin, with a 95% confidence level and 5% precision, and assuming an expected prevalence of 20% (derived from a pilot study conducted by the researchers and which included 124 patients), a sample size of 246 patients was determined to be necessary.

### 4.2. Data Collection

The patients’ data were retrieved from the GHAF^®^, SClinico^®^, and Modulab^®^ hospital platforms (accessed between 24 March 2023 and 4 August 2023) for the Local Health Unit of Guarda. Similarly, data were gathered from the Glintt^®^—Integrated Management System of the Medicine Circuit and SClinico^®^ hospital platforms (accessed from 24 June 2023 to 30 June 2023) for the Local Unit Health of Cova da Beira. The following variables were analyzed:

(a) Age and gender.
(b) Medical history: T2DM, high blood pressure (HBP), heart failure (HF), and chronic kidney disease (CKD).
(c) Blood glycemia, creatinine, sodium, and potassium levels [both before dapagliflozin prescription (baseline) and 5 to 8 days after prescription (endpoint)]. The exact endpoint day (between day 5 and day 8 after dapagliflozin prescription) was determined following the KDIGO guidelines, which define AKI as an abrupt decrease in kidney function occurring within 7 days [62]. Due to limitations in the data availability, most of the hospitalized patients did not have analytical parameters available daily or on weekends.
(d) Individual Cases Safety Reports (ICSRs) data were obtained from the European spontaneous reporting system EV database, accessed at www.adrreports.eu (accessed on 7 February 2024). The EV, funded by the European Medicines Agency EMA, manages and analyses the ICSRs for suspected ADRs [63,64].
I. Qualitative and quantitative analyses of the main outcomes of the ICSRs were conducted from 1 January to 31 December, 2023.
II. The information collected included sex, age group, outcomes, number of AKIs, hyponatremia, and hypokalemia events per ICSR, as well as reported concomitant medications (furosemide, ACEi, ARB, or spironolactone).

### 4.3. Statistical Analysis

The statistical analysis was performed using IBM SPSS statistics 28 (IBM, Armonk, NY, USA). The categorical variables were described using absolute and relative frequencies (percentages). A Wilcoxon signed-rank test, Pearson’s chi-square test, and Fisher’s exact test were used, with a statistical significance level set at 5% (*p* < 0.05).

## 5. Conclusions

This retrospective study provides valuable insights into the short-term effects of dapagliflozin on renal function and electrolyte balance in patients with T2DM. Dapagliflozin demonstrated effectiveness for reducing blood glucose levels within the initial days of treatment, affirming its role as a rapid-acting antidiabetic agent. However, concerns regarding its renal safety were evident, with a significant increase in blood creatinine levels post-dapagliflozin administration, necessitating the close monitoring of renal function in clinical practice, particularly in the first few weeks after starting dapagliflozin.

Electrolyte disturbances, particularly hyponatremia and hyperkalemia, were prevalent among the patients receiving dapagliflozin, emphasizing the importance of electrolyte monitoring and risk management strategies. The concomitant use of loop diuretics, such as furosemide, was associated with a higher incidence of acute kidney injury, highlighting the intricate relationship between SGLT2 inhibitors and diuretic therapy.

Although our study did not find a significant association between dapagliflozin use and electrolyte imbalances in the patients with chronic kidney disease, the management of comorbidities remains a clinical challenge. The continuous surveillance of renal function and electrolyte levels is imperative to mitigate the risk of adverse events and to optimize treatment outcomes for this vulnerable patient population.

In conclusion, our real-world data contribute to a better understanding of the clinical implications of dapagliflozin therapy, informing evidence-based decision making and enhancing the quality of diabetes care. Further research is warranted to elucidate the long-term safety and efficacy of dapagliflozin across diverse patient populations and clinical contexts, with an emphasis on tailored treatment approaches and comprehensive patient management.

## Figures and Tables

**Table 1:** Sample characterization.

Age (Mean ± Std)	78.70 ± 10.72
Comorbidities	
Type 2 diabetes mellitus	149 (60.6%)
High blood pressure	185 (75.2%)
Heart failure	114 (46.3%)
Chronic kidney disease	64 (26.0%)
Concomitant medication	
Furosemide	174 (70.7%)
Spironolactone	94 (38.2%)
Angiotensin-converting enzyme inhibitors	88 (35.8%)
Angiotensin receptor blockers	86 (35.0%)
Potassium supplementation	77 (31.7%)

**Table 2:** Blood creatinine levels and acute kidney injury (*n* = 246).

Blood Creatinine (mg/dL)	Mean ± Std	Min–Max	*p*-Value
Baseline	1.26 ± 0.59	0.49–4.18	0.0001 ^1^
Endpoint	1.39 ± 0.77	0.39–6.55	
Creatinine increase ≥ 0.3 mg/dL (*n* = 48)			
Gender	Male	28 (50.9%)	0.878 ^2^
	Female	27 (49.1%)	
Age (years)	<65	4 (7.3%)	0.232 ^2^
	65–75	8 (14.5%)	
	76–85	22 (40.0%)	
	>85	21 (38.2%)	

^1^ Wilcoxon signed-rank test; ^2^ Pearson’s chi-square test.

**Table 3:** Blood sodium levels at baseline and at the endpoint (*n* = 246).

Blood Sodium (mEq/L)	Mean ± Std				Min–Max	*p*-Value
Baseline	139.05 ± 4.52				119–158	0.0009 ^1^
Endpoint	138.48 ± 5.36				115–161	
	Baseline (*n*; %)	T2DM (*n*; %)	HBP (*n*; %)	HF (*n*; %)	CKD (*n*; %)	
<135	25 (10.2%)	12 (4.9%)	20 (8.1%)	9 (3.7%)	11 (4.5%)	
135–145	213 (86.6%)	133 (54.1%)	161 (65.4%)	102 (41.5%)	51 (20.7)	
>145	8 (3.3%)	4 (1.6%)	4 (1.6%)	3 (1.2%)	2 (0.8%)	
	Endpoint (*n*; %)					0.0146 ^2^
<135	43 (17.5%)	26 (10.6%)	32 (13.0%)	26 (10.6%)	14 (5.7%)	
135–145	188 (76.4%)	112 (45.5%)	140 (56.9%)	83 (37.3%)	47 (19.1%)	
>145	15 (6.1%)	11 (4.5%)	13 (5.3%)	5 (2.0%)	3 (1.2%)	

^1^ Wilcoxon signed-rank test; ^2^ Pearson’s chi-square test.

**Table 4:** Blood potassium levels at the baseline and at the endpoint (*n* = 246).

Blood Potassium (mmol/L)	Mean ± Std	Min–Max				*p*-Value
Baseline	4.19 ± 0.60	2.5–6.2				<0.0001 ^1^
Endpoint	4.44 ± 0.62	2.8–6.4				
	Baseline (*n*; %)	T2DM (*n*; %)	HBP (*n*; %)	HF (*n*; %)	CKD (*n*; %)	
<3.5	28 (11.4%)	13 (5.3%)	24 (9.8%)	14 (5.7%)	4 (1.6%)	0.0002 ^2^
3.5–5.0	196 (79.7%)	125 (50.8%)	144 (58.5%)	92 (37.4%)	55 (22.4%)	
>5.0	22 (8.9%)	11 (4.5%)	17 (6.9%)	8 (3.3%)	5 (2.0%)	
	Endpoint (*n*; %)					
<3.5	8 (3.3%)	4 (1.6%)	5 (2.0%)	3 (1.2%)	1 (0.4%)	
3.5–5.0	197 (80.1%)	16 (51.2%)	149 (60.6%)	96 (39.0%)	50 (20.3%)	
>5.0	41 (16.7%)	19 (7.7%)	31 (12.6%)	15 (6.1%)	13 (5.3%)	
Blood potassium at the endpoint	≤5 mmol/L (*n*; %)	>5 mmol/L (*n*; %)				*p*-value
Potassium supplementation	61 (79.2%)	16 (20.8%)				0.243 ^2^
ACEi	78 (88.6%)	10 (11.4%)				0.096 ^2^
ARB	65 (75.6%)	21 (24.4%)				0.017 ^2^
Spironolactone	74 (78.7%)	20 (21.3%)				0.149 ^2^

^1^ Wilcoxon signed-rank test; ^2^ Pearson’s chi-square test.

**Table 5:** Individual Cases Safety Reports analysis from 1 January 2023 to 31 December 2023.

Individual Cases Safety Reports	2666 (100%)			
Sex				
Male	1517 (56.9%)			
Female	1033 (38.7%)			
Not Specified	116 (4.4%)			
Age group				
16–64	612 (23.0%)			
65–85	1017 (38.1%)			
>85	199 (7.5%)			
Not specified	833 (31.2%)			
	Use			
Individual cases reported by system organ classes	Total	Chronic Kidney Disease 237 (8.9%)	Diabetes Mellitus 1425 (53.5%)	Heart Failure 502 (18.9%)
Renal and urinary disorders	394 (14.8%)	53 (13.5%)	226 (57.4%)	113 (28.7%)
Cardiac disorders	126 (4.7%)	6 (4.8%)	41 (32.5%)	46 (36.5%)
Gastrointestinal disorders	314 (11.8%)	25 (8.0%)	137 (43.6%)	56 (17.8%)
Infections	595 (22.3%)	33 (5.5%)	303 (50.9%)	122 (20.5%)
Concomitant Drug/Adverse Drug Reaction				
	Acute Kidney Injury (*n* = 98)	*p*-value	Hyponatremia (*n* = 21)	*p*-value
Furosemide (*n* = 120)	5 (4.2%)	0.770 ^1^	3 (2.5%)	0.066 ^2^
	Hyperkaliemia (*n* = 17)		*p*-value	
Spironolactone (*n* = 89)	4 (4.5%)		0.002 ^2^	
ACEi (*n* = 186)	2 (1.1%)		0.335 ^2^	
ARA (*n* = 364)	2 (0.5%)		1.000 ^2^	

^1^ Pearson’s chi-square test; ^2^ Fisher exact test.

## Data Availability

The datasets generated during and/or analyzed during the current study are available from the corresponding author upon reasonable request.

## Appendix A

**Table A1:** Relationship between dapagliflozin and furosemide administration and acute kidney injury.

	Furosemide		*p*-Value
	No	Yes	
Without AKI at endpoint	64 (33.5%)	127 (66.5%)	0.006 ^1^
AKI at endpoint	8 (14.5%)	47 (85.5%)	

^1^ Pearson’s chi-square test.

**Table A2:** Relationship between CKD, hyponatremia, and hyperkalemia.

	Chronic Kidney Disease		*p*-Value
	No	Yes	
Hyponatremia	34 (66.7%)	17 (33.3%)	0.181 ^1^
Hyperkaliemia	28 (68.3%)	13 (31.7%)	0.363 ^1^

^1^ Pearson’s chi-square test.
